# Significant Surface Spin Effects and Exchange Bias in Iron Oxide-Based Hollow Magnetic Nanoparticles

**DOI:** 10.3390/nano12030456

**Published:** 2022-01-28

**Authors:** Pelayo García Acevedo, Manuel A. González Gómez, Ángela Arnosa Prieto, Jose S. Garitaonandia, Yolanda Piñeiro, José Rivas

**Affiliations:** 1 NANOMAG Laboratory, Applied Physics Department, iMATUS Materials Institute, Universidade de Santiago de Compostela, 15782 Santiago de Compostela, Spain; manuelantonio.gonzalez@usc.es (M.A.G.G.); angela.arnosa@usc.es (Á.A.P.); y.pineiro.redondo@usc.es (Y.P.); 2 Faculty of Science and Technology, University of the Basque Country (UPV/EHU), 48940 Leioa, Spain; js.garitaonandia@ehu.eus

**Keywords:** magnetic nanoparticles, nanomagnetism, exchange bias, surface spins, interfacial effects

## Abstract

Exchange bias (EB) properties have become especially important in hollow magnetic nanoparticles (MNPs) due to the versatility and reduced size of these materials. In this work, we present the synthesis and study of the EB properties of iron-oxide-based hollow MNPs and their precursors Fe/iron oxide MNPs with core/void/shell structure. The two mechanisms involved in EB generation were investigated: the frozen spins present in the nanograins that form the nanoparticles and the surface spins. The effect of external parameters on the coercivity (H_C_), remanence (M_R_), exchange bias field (H_EB_) and frozen spins, such as cooling field (H_FC_) and temperature, was investigated. Both H_C_ and H_EB_ present a maximum threshold above which their values begin to decrease with H_FC_, showing a new trend of H_EB_ with H_FC_ and allowing modulation on demand. The existence of surface spins, present on the outer and inner surfaces, was demonstrated, and an intrinsic EB phenomenon (H_EB_ = 444 Oe for hollow iron oxide-based MNPs of 13.1 nm) with significant magnetization (M_S_~50 emu/g) was obtained. Finally, core/void/shell MNPs of 11.9 nm prior to the formation of the hollow MNPs showed a similar behavior, with non-negligible H_EB_, highlighting the importance of surface spins in EB generation.

## 1. Introduction

Exchange bias is a magnetic phenomenon in which magnetic phases of different character are coupled via exchange interaction and their magnetic behavior departs from a simple response, giving rise to a shift from the zero-field position of the hysteresis loop at low temperatures. Although interest in EB is growing, owing to its technological applications in the electronic field (spintronic devices [1], spin valves [2], magnetoresistive random-access memory (MRAM) circuits [3]), or new recording media [4] based on materials where the superparamagnetic limit could be defeated with the help of EB-tailored properties, the detailed understanding of EB is still poorly understood.

Initially, the prototypical magnetic system showing EB was a ferromagnetic (FM)/antiferromagnetic (AFM) binary system, cooled through the Néel temperature under the application of an external magnetic field [5,6,7]. Subsequently, it was further observed in other binary systems at the interface of ferromagnetic/ferrimagnetic (FM/FI) [8] or antiferromagnetic/ferrimagnetic (AF/FI) [9] materials. Despite this, most efforts have been focused on studying the EB phenomenon in thin-layer systems; and since its observation in MNPs in 1956 [10], the interest in EB in nanomaterials has increased. In recent years, the improvement of MNP synthesis, which allows the production of MNPs with strict size and shape control, has turned relevant MNPs into interesting tools to study fundamental EB mechanisms and alternative solutions for small size applications. In fact, the possibility to produce solid cores, core@shell, and hollow nanostructures allows the study of the cross-over contribution of a different set of mechanisms related to EB: surface spin disorder and exchange coupling of different magnetic phases.

The impact of size reduction on the magnetic properties of solid MNPs, caused by the large contribution of a disordered surface spin layer, has been widely studied for different materials, such as CoFe_2_O_4_ MNPs of 4.5 nm (H_EB_ = 735 Oe) [11] or NiO MNPs of 4 nm (H_EB_ = 900 Oe) [12], and, more intensely, in γ-Fe_2_O_3_ MNPs [13,14,15,16]. EB observed in solid MNPs is attributed to the exchange coupling between disordered surface spins that may become frozen in a spin-glass-like state and inner-ordered spins [5]. In Fe oxide-based MNPs, significant differences in magnetization have been found between small (5 nm) Fe_3_O_4_ and γ-Fe_2_O_3_ MNPs [17,18], owing to the notable contribution of surface effects at this size scale and the fact that surface spin disorder is stronger in γ-Fe_2_O_3_ MNPs than in Fe_3_O_4_ MNPs [5]. Although EB effects, ascribed to exchange coupling between the disordered surface and antiferromagnetically ordered structure of the core [19], have been observed in γ-Fe_2_O_3_ MNPs, and also in Fe_3_O_4_ MNPs, their detailed understanding in terms of size remains elusive owing to contradictory reported data. Systematic studies of the size effects related to EB in small Fe_3_O_4_ MNPs, of 4, 6, 8, 10, and 12 nm, only report EB effects (H_EB_ = 750 Oe) in the smaller samples (4 nm) [18]. Equally sized small (8 nm) Fe_3_O_4_ and γ-Fe_2_O_3_ MNPs [20] show larger and stronger surface disorder in γ-Fe_2_O_3_ and should provide higher H_EB_. Moreover, as the size of γ-Fe_2_O_3_ MNPs increases, 7 nm [21], 8 nm [20], 10 nm [22], they show an increasing EB, H_EB_ = 60 (Oe), H_EB_ = 267 (Oe), H_EB_ = 1500 (Oe), respectively, for decreasing surface spin. Although surface effects and magnetic frustration are more noticeable as the size of MNPs is reduced, a defined relationship between size and H_EB_ cannot be established from the data reported in previous research, since EB depends in a complex way on other factors, such as shape or magnetic interactions [5]. Besides this, since EB in solid MNPs requires surface spin effects that become relevant in MNPs with a large size reduction, the consequence of this entails a reduction in magnetization [18]. Therefore, new types of MNP with EB properties, such as core/shell MNPs with a coupling between the core and the shell [23], or hollow MNPs, with an additional layer of surface spins contributing to increased spin disorder [24], can be envisaged as alternative systems showing EB and moderate magnetization simultaneously.

Core/shell MNPs, such as thin-layer systems, have been reported to show EB, mainly due to the exchange coupling between different magnetic materials: Fe/γ-Fe_2_O_3_ with particle sizes of 15 nm (H_EB_ = 1.6 kOe), 10 nm (H_EB_ = 3.5 kOe) and 8 nm ( H_EB_ = 3200 Oe) [25]; Fe/Fe_3_O_4_ MNPs of 13.8 nm (H_EB_ = 1190 Oe) [23]; FeO/Fe_3_O_4_ MNPs of 10 nm (H_EB_ = 1700 Oe) [26]; Fe_3_O_4_/γ-Fe_2_O_3_ MNPs of 12 nm (H_EB_ = 270 Oe) [27] or CoO/Fe_3_O_4_ MNPs [28]. Different magnetite-based systems, combining an antiferromagnetic core (FeO) and a ferrimagnetic shell (Fe_3_O_4_) in a wide range of sizes (10–35 nm) [26,29,30,31], have been explored to produce tailored EB (Xiaolian Sun et al. [30] have reported H_EB_ = 2260 Oe in FeO/Fe_3_O_4_ MNPs of 35 nm). However, in addition to the core/shell coupling, the surface effects become relevant and even dominant in EB. In fact, a critical particle size in Fe/γ-Fe_2_O_3_ MNPs, in which the interface spin effect contributes primarily to the EB, but below which the surface spin effect dominates, has been established by keeping the shell thickness (2 nm) fixed and varying the core size (4–11 nm) [25]. However, different studies aiming to assess the importance of surface spins in the EB of core/shell NPs provide contradictory results. On one hand, it was stated that no substantial modification of H_EB_ was observed between the core/shell and void/shell system after removing the core in Fe/γ-Fe_2_O_3_ MNPs [32], while in different works, a drastic reduction in H_EB_ was observed in core/shell MNPs after keeping only the Fe_3_O_4_ shell, showing the low importance of surface spins in this type of systems [5,23,33].

The transformation from core/shell MNPs to hollow MNPs using the Kirkendall effect [33], is a useful chemical tool to create nanostructures with tailored surface–volume ratios to further analyze EB in hollow MNPs. Although EB in hollow MNPs was firstly attributed only to the large portion of disordered spins located in the inner and outer shells [5], other studies show that the interfaces between the crystallographic domains of MNPs may also play an important role [34]. Although H_EB_ has been studied in hollow MNPs with diverse compositions, such as CoFe_2_O_4_ (H_EB_ = 733 Oe) [35], NiFe_2_O_4_ (H_EB_ = 301 Oe) [36] or Fe_3_O_4_ (H_EB_ = 133 Oe) [23], studies on γ-Fe_2_O_3_ hollow MNPs are the most abundant [13,24,34,37]. A large hysteresis loop shift has been reported in hollow γ-Fe_2_O_3_ as small as 8.2 nm [37], 9.2 nm [34], and 9.4 nm [32]. However, since the maximum applied field is smaller than the irreversibility field, these shifts correspond to a minor loop and not an intrinsic EB effect. In addition, a significant loop shift (7000 Oe) was also reported in larger γ-Fe_2_O_3_ hollow MNPs (14.7 nm) [32]. On the other hand, an intrinsic EB phenomenon has been reported in larger γ-Fe_2_O_3_ hollow MNPs (18.7 nm) [34]. However, the H_EB_ obtained is significantly lower than the loop shifts obtained in ultrathin hollow γ-Fe_2_O_3_ with minor loop effects. Large differences were reported between the surface spin contributions above the magnetic properties in hollow γ-Fe_2_O_3_ MNPs of 9 and 18 nm, obtaining a percentage of surface spins of 87 and 3% for the γ-Fe_2_O_3_ MNPs of 9 nm (minor loop) and 18 nm (EB), respectively [32]. This suggests that a greater presence of surface spins contributes significantly to minor loop generation and not to EB. However, interestingly, F. Sayed et al. [38] recently reported the existence of an intrinsic EB effect in ultrathin hollow γ-Fe_2_O_3_ MNPs (9.4 nm). From Mossbauer experiments, the disorder of surface spins was shown and through Monte Carlo simulations, the fundamental role of the surface anisotropy in the noncollinearity of the spin structure was corroborated. In addition, F. Sayed et al. [24] reported a computer model of hollow MNPs as a function of size and shell thickness. The influence of the shell thickness of hollow MNPs on the spin configuration was reported, with the conclusion that the superficial anisotropy (from both the internal and external surfaces) becomes larger as the shell diminishes. In addition, a H_EB_ = 133 Oe value in 16.0 nm hollow Fe_3_O_4_ MNPs was reported by Ong et al. [23], the only recent study to report EB in hollow Fe_3_O_4_ MNPs. However, due to the small size of hollow γ-Fe_2_O_3_ (<10 nm), the surface effects led to a significant magnetization reduction of MNPs. In addition, large differences between the minor loop effect and intrinsic EB were observed in hollow MNPs. Therefore, more experimental studies of hollow MNPs with an intrinsic EB and with significant magnetization to improve its application should be carried out.

Exchange bias generation in MNPs is affected by several factors, such as particle size, the thickness of the shell in core/shell MNPs or in hollow MNPs, temperature, cooling field (H_FC_), morphology, and composition [5]. Especially important is the effect of temperature and H_FC_ on modifying EB, since it is an externally controllable factor in obtaining H_EB_ on demand. Temperature dependence has been widely demonstrated, showing a reduction in the EB effect with increasing temperature [5,21,22,25,32,39]. Despite the numerous EB studies reported on MNPs, there is no well-defined trend in H_EB_ dependence with H_FC_. While in Fe/Fe_3_O_4_ MNPs, H_EB_ grows with H_FC_ (H_FC_^Max^ = 10 kOe) [23], in CoFe_2_O_4_ MNPs an H_EB_ maximum has been observed at H_FC_ = 5 kOe (lower than H_FC_^Max^ = 15 kOe) [40]. A similar trend was observed in Fe_3_O_4_ MNPs. However, the H_EB_ maximum was reported at a higher H_FC_ (30 kOe) [41]. In addition, H_EB_ dependence with H_FC_ up to 50 kOe in a granular system of Fe nanoparticles was investigated, showing a H_EB_ maximum at 4 kOe [42]. Despite the multiple efforts made in EB generation in hollow γ-Fe_2_O_3_ MNPs, to our knowledge, no studies of H_EB_ dependence with H_FC_ have been reported. It is therefore necessary to carry out more studies in this area.

Numerous studies have reported a hysteresis loop shift in hollow iron-oxide-based MNPs. However, many have been attributed to a minor loop effect and not an intrinsic effect of EB, especially in hollow γ-Fe_2_O_3_ MNPs. In addition, due to the small size of hollow γ-Fe_2_O_3_ MNPs (<10 nm), the surface effects lead to a significant magnetization reduction of MNPs and the maximum magnetization of these MNPs is very low (9.2 nm, M_MAX_ < 1 emu/g [38]; 9.2 nm, M_MAX_~1 emu/g [34]; 9.4 nm, M_MAX_~1.2 emu/g [32]; 8.2 nm, M_MAX_~6 emu/g [37]). These combinations of minor loops instead of intrinsic EB and reduced magnetization may be a drawback in the use of hollow γ-Fe_2_O_3_ MNPs for several applications. Therefore, in order to gain insights on EB in hollow γ-Fe_2_O_3_ or Fe_3_O_4_ MNPs, systematic studies are required to assess different aspects of H_EB_ and its dependence with extended values of H_FC_, since the current field is very limited.

In the present work, an intrinsic EB effect (H_EB_ = 444 Oe) in iron oxide-based hollow MNPs of 13.1 nm with a significant magnetization (around 50 emu/g) is studied in detail. The dependence of H_C_, M_R_ and H_EB_ with H_FC_ and temperature using H_FC_ up to 50 kOe, as well as the variation in the number of frozen spins with H_FC_ and temperature, were investigated to shed light on the field-cooling effects on EB, producing new results not reported so far in iron oxide hollow MNPs. In addition, the presence of surface spins as a new way to improve the EB properties of hollow MNPs was studied. To understand in depth the EB generation mechanism, a detailed study of the magnetic properties of the precursor core/void/shell MNPs (with Fe core) was performed prior to the complete oxidation of the Fe core, allowing us to shed light on EB generation in hollow iron-oxide-based MNPs.

## 2. Materials and Methods

### 2.1. Chemicals and Materials

Chemicals used for this study were iron (0) pentacarbonyl (Fe(CO)_5_, >99.99%), oleylamine (C_18_H_35_NH_2_, 80–90%), 1-octadecene (C_18_H_36_, 90%), trimethylamine N-oxide ((CH_3_)_3_NO, 98%), and hexane(CH_3_(CH_2_)_4_CH_3,_ >95%), all obtained from Sigma-Aldrich (Burlington, MA, USA).

### 2.2. Synthesis of Core/Void/Shell Nanoparticles (Fe/Iron Oxide)

MNPs were obtained following Peng’s method [43] with some modifications. In a typical synthesis, 1-octadecene (200 mL) and oleylamine (3 mL, 9.12 mmol) were degassed under nitrogen at 120 °C for 3 h to eliminate the oxygen of the mixture. After that, the solution was heated until 180 °C and iron pentacarbonyl (7 mL, 53.2 mmol) was quickly added under nitrogen. The reaction was kept at 180 °C for 40 min. The mixture was cooled naturally to room temperature.

### 2.3. Synthesis of Hollow Magnetic Nanoparticles (Iron Oxide)

Hollow MNPs were prepared by adding trimethylamine N-oxide (400 mg) and 1-octadecene (200 mL) in a three-necked round flask. The mixture was degassed with nitrogen throughout the procedure and mechanically stirred. The reaction was heated at 130 °C for 1 h, followed by quickly adding core/void/shell (C/V/S) MNPs (800 mg) dispersed in hexane into the mixture and heated for 2 h to remove the hexane and it was heated at 210 °C for 4 h. The product was allowed to cool down to room temperature.

Oleylamine was used in both MNPs as a functional coating in order to avoid direct interactions between the MNPs.

### 2.4. Physicochemical and Magnetic Characterization of Core/Void/Shell and Hollow MNPs

The characterization of the crystalline phases of the MNPs was performed by X-ray diffraction (XRD) on powder samples with a Philips PW1710 diffractometer (Panalytical, Brighton, UK) and a Cu K_a_ radiation source, λ = 1.54186 Å. Measurements were collected in the 2θ angle range between 10 and 80° with steps of 0.02° and 10 s per step. Morphology of the MNPs was characterized by transmission electron microscopy (TEM) images using a JEOL JEM-1011 microscope (JEOL, Tokyo, Japan) at 100 kV. Iron content of the MNPs was determined by flame atomic absorption spectroscopy (FAAS) in a Perkin Elmer 3110 Atomic Absorption Spectrometer (Waltham, MA, USA). Fourier transform infrared (FTIR) spectra were recorded in a Thermo Nicolet Nexus spectrometer (Thermo Fisher Scientific, Madrid, Spain) using the attenuated total reflectance (ATR) method. Mössbauer spectroscopy measurements were performed at room temperature in transmission geometry using a conventional constant-acceleration spectrometer with ^57^Co-Rh source. The isomer shift values were taken with respect to an α-Fe calibration foil measured at room temperature. NORMOS Mössbauer fitting program (version 16.07.2001) developed by Brand et al. [44] was used for fitting the spectra. AC magnetization curves of dried samples were measured using a Quantum Design Physical Property Measurement System (PPMS) (Quantum Design, Darmstadt, Germany) in a temperature range of 10–300 K with steps of 10 K, an excitation field of 1 Oe and driving frequencies varying from 10 Hz to 1 kHz. DC magnetization curves of dried samples were measured using a Superconducting Quantum Interference Device (SQUID) Magnetometer (Quantum Design, Darmstadt, Germany). Measurements of the MNPs magnetization were made in field-cooled (FC) and zero-field-cooled (ZFC) conditions employing different cooling fields (H_FC_ = 0.1–50 kOe) and as a function of temperature (T = 5–300 K). The effect of temperature and applied cooling field on the hysteresis loops (H = ±50 kOe) for the MNPs was analysed.

## 3. Results and Discussion

### 3.1. Structural, Chemical, and Morphological Properties

#### 3.1.1. XRD Characterization

**Figure 1:**
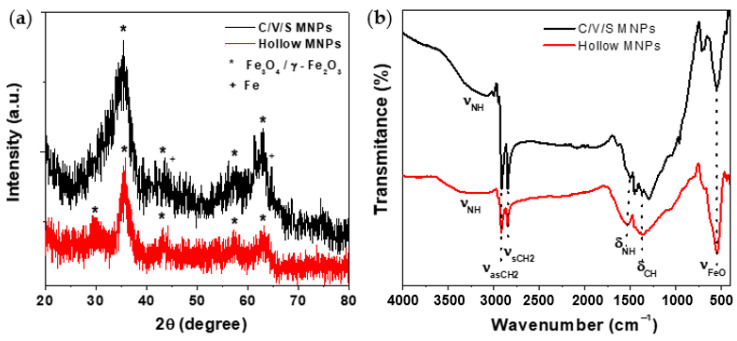
(**a**) XRD pattern of C/V/S (black pattern) and hollow (red pattern) MNPs, compared to the XRD pattern of magnetite/maghemite (*) from JCPDS 79-0417 and iron (+) from JCPDS 89-4184 data bases. (**b**) FT-IR spectra of C/V/S (black pattern) and hollow (red pattern) MNPs.

Figure 1a shows the powder XRD patterns of the C/V/S (black pattern) and the hollow MNPs (red pattern). The position and relative intensities of the main peaks could indicate the presence of magnetite with inverse spinel structure (JCPDS card No. 79-0417) [45]. However, this iron oxide phase could also be associated with maghemite, since these two oxides cannot be distinguished from XRD. The magnetite and maghemite have almost identical or closely crystal structures. Furthermore, 110 and 200 reflections from iron with cubic structure can be observed in the C/V/S MNPs (JCPDS card No. 89-4184) [46].

A lower crystallinity was observed in C/V/S MNPs due to the greater width of the main peak of the XRD pattern. This fact is consistent with the synthesis process of MNPs described by Peng et al. [43], which started from a completely amorphous core/shell MNPs before the crystallinity increased as the C/V/S MNPs were transformed into hollow MNPs. In this study, it was observed that C/V/S MNPs are in an intermediate state of crystallinity between completely amorphous core/shell MNPs and crystalline hollow MNPs. This made it possible to show that in C/V/S MNPs, the oxidation process of the Fe core begins prior to the complete oxidation of the Fe core and the formation of the hollow iron oxide MNPs.

#### 3.1.2. FT-IR Spectroscopy

The FT-IR spectra of the hollow MNPs and the C/V/S precursor MNPs are shown in Figure 1b, black pattern (C/V/S MNPs) and red pattern (hollow MNPs). The FTIR spectra of both samples show a broad absorption band around 3327 cm^−1^, which is characteristic of the stretching vibrations of the O-H group attributed to the presence of hydroxyl residue, which is due to atmospheric moisture [47]. In addition, the peaks appearing at 2920 and 2849, and 1550 and 1305 cm^−1^, are assigned to the stretching vibrations of -CH_2_ (asymmetric and symmetric) and the scissoring vibrations (N-H and -C-H) of oleylamine [48], respectively. This oleylamine coating makes it possible to avoid direct interactions between MNPs and their effect on EB generation. Furthermore, the peak at about 550 cm^−1^ is characteristic of the stretching vibration of Fe_3_O_4_ [49].

#### 3.1.3. Transmission Electron Microscopies (TEM)

**Figure 2:**
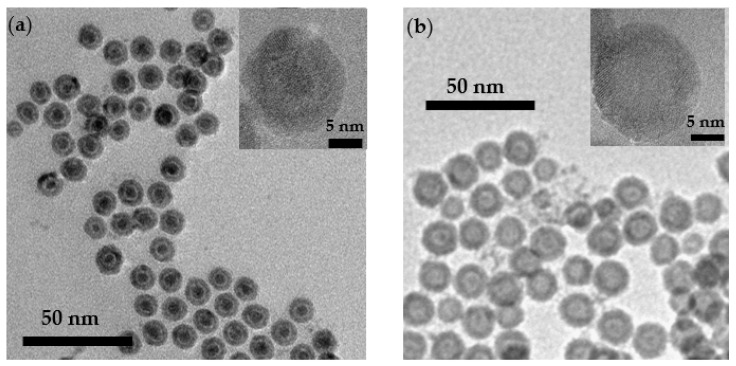
TEM micrograph of (**a**) core/void/shell and (**b**) hollow iron oxide MNPs dispersed in hexane. The size distribution was performed using the ImageJ software and shows particle sizes of 11.9 ± 0.9 nm for core/void/shell MNPs and 13.2 ± 0.7 nm for hollow iron oxide based MNPs. Insets shows HRTEM images of MNPs.

Figure 2 shows TEM micrographs of (a) the precursor C/V/S and (b) the hollow MNPs. The insets show high-resolution TEM images (HRTEM). A regular spherical morphology with a relatively wide size distribution of around 11.9 ± 0.9 nm (C/V/S MNPs) and 13.2 ± 0.7 nm (hollow MNPs) was obtained. A void space can be observed between the Fe core and the iron oxide shell, suggesting a core/void/shell morphology. On the other hand, in Figure 2b, the hollow morphology of the MNPs can be observed due to the darker color of the shell.

From the HRTEM and TEM micrographs it is possible to obtain the shell thickness, as well as the inner and outer radius of both MNPs. Table 1 shows the morphological values of the MNPs. Shell thicknesses of 2.4 ± 0.3 nm for C/V/S MNPs and 3.7 ± 0.3 nm for hollow MNPs were obtained, showing a shell increase. Furthermore, a Fe core size of 4.5 ± 0.5 nm and a void between the core and the shell of 1.3 ± 0.2 nm were obtained in C/V/S MNPs. From these values, the surface-to-volume (S/V) ratio can be obtained from the following expressions:

(1) $$R_{\mathrm{Hollow}}=\frac{S}{V_{\mathrm{shell}}}=\frac{S_{\mathrm{Outer}}+{\;S}_{\mathrm{inner}}}{V_{\mathrm{Total}}-V_{\mathrm{inner}}}\;$$

(2) $$R_{C/V/S}=\frac{S}{V_{\mathrm{shell}}+V_{\mathrm{Core}}}=\frac{S_{\mathrm{Outer}}+{\;S}_{\mathrm{inner}}+{\;S}_{\mathrm{core}}}{(V_{\mathrm{Total}}-V_{\mathrm{inner}})+V_{\mathrm{Core}}}\;$$

where S_outer_ and S_inner_ correspond to the outer and inner surface of the shell and S_core_ and V_core_ correspond to the surface and volume of the Fe core, respectively.

The surface-to-volume ratio is higher for the C/V/S MNPs (S/V = 0.9) than for the hollow MNPs (S/V = 0.6), due to the smaller size of the C/V/S MNPs and the contribution of the Fe core surface. This suggests greater disorder and magnetic frustration in the C/V/S MNPs.

#### 3.1.4. Room Temperature Mössbauer Spectra

**Figure 3:**
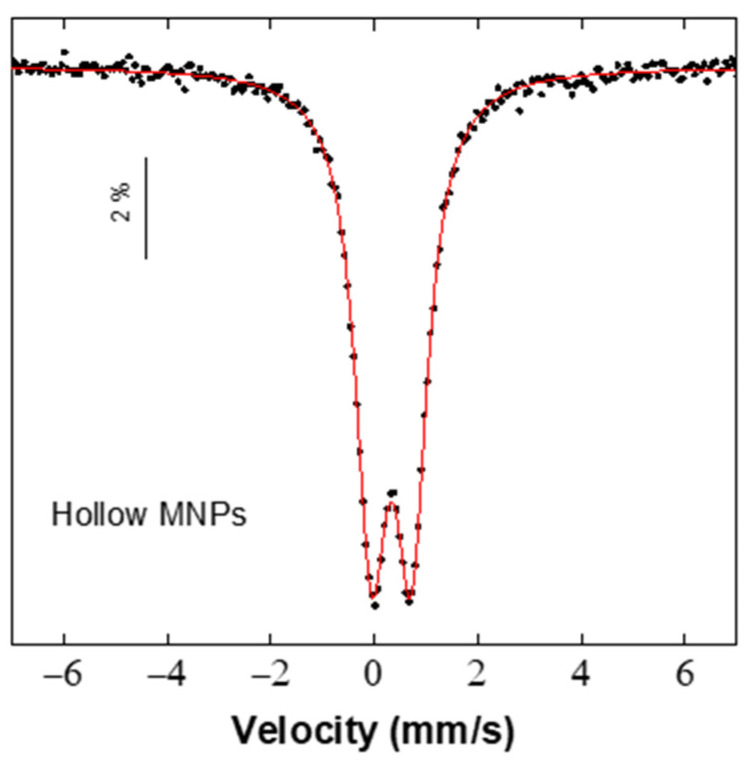
Room temperature ^57^Fe Mössbauer spectrum of superparamagnetic hollow iron oxide MNPs.

Figure 3 shows the Mössbauer spectrum corresponding to the hollow iron oxide MNPs obtained at room temperature.

The spectrum of hollow MNPs is characterized by a well resolved doublet and the absence of other any additional component, as expected from samples composed by homogenously sized MNPs well above the blocking temperature (T_B_) in a superparamagnetic regimen. Hyperfine parameters, with an isomer shift (IS) of 0.33 mm/s, quadrupolar splitting (QS) of 0.76 mm/s, and a linewidth of 0.75 mm/s were obtained for the hollow iron oxide MNPs. Spectra with these hyperfine parameters are commonly observed in systems composed by superparamagnetic magnetite-precursor MNPs [50] and in low-dimensional magnetite MNPs systems with high surface/core ratio [51,52].

### 3.2. DC Magnetic Properties

**Figure 4:**
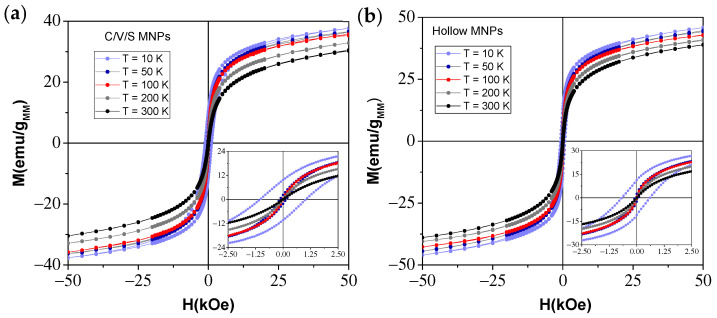
Hysteresis loops of (**a**) core/void/shell MNPs and (**b**) hollow iron oxide MNPs at different temperatures: 10, 50, 100, 200 and 300 K. Insets: Scale amplification of hysteresis loops.

In order to understand the DC magnetic properties of the C/V/S MNPs and hollow MNPs, hysteresis loops were performed as the temperature reduced from 300 to 10 K. Figure 4 shows the hysteresis loops of the C/V/S MNPs (Figure 4a) and the hollow MNPs (Figure 4b) at different temperatures: 10 K (light blue), 50 K (dark blue), 100 K (red), 200 K (gray), and 300 K (black). These were performed with a SQUID magnetometer between −50 and +50 kOe. The insets show the low-field region in more detail, where the coercivity and remanence of both samples can be observed. Neither MNP presented saturation, despite the use of very high fields, ±50 kOe, and a noticeable paramagnetic contribution can be observed in the hysteresis loops.

Maximum magnetization values at H = 50 kOe between 38.17 (10 K) and 30.75 (300 K) emu/g and 45.93 (10 K) and 38.90 (300 K) emu/g were obtained for the C/V/S and hollow MNPs, respectively. Lower values of magnetization were observed in C/V/S MNPs due to lower crystallinity than in hollow MNPs. However, the magnetization values did not differ too much between both MNPs. This agrees with the XRD results, from which it can be concluded that the C/V/S were in an intermediate state of crystallinity prior to the formation of hollow MNPs. Despite the high values of the S/V ratio, the magnetization was not drastically reduced by surface effects. This suggests that hollow MNPs may emerge as an interesting alternative, presenting improved magnetic properties and exhibiting an intrinsic EB effect.

### 3.3. Exchange Bias Properties

**Figure 5:**
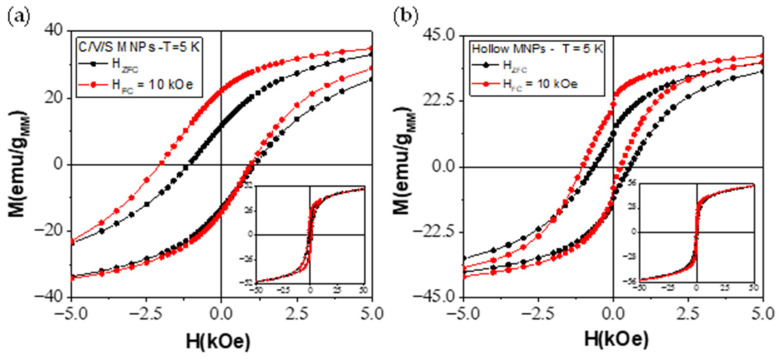
Hysteresis loops of (**a**) core/void/shell MNPs and (**b**) iron oxide hollow MNPs at 5 K cooled without field (ZFC, black points) and with H_FC_ = 10 kOe (FC, red points) in the range of −50 kOe and 50 kOe. For clarity only the range between −5 kOe and 5 kOe is shown. Insets: Complete hysteresis loops. A hysteresis loop shift is observed in both X (field) and Y (magnetization) axes, indicating the presence of EB.

In order to study the EB and spin disorder properties, the magnetization of the specimens as a function of magnetic field and temperature was measured under FC, using 11 different cooling fields (0.1–50 kOe) and ZFC conditions. All the hysteresis loops were performed using the same measuring field (between −50 kOe and 50 kOe). Figure 5 shows hysteresis loops with ZFC conditions (black points) and with H_FC_ = 10 kOe (red points) for C/V/S MNPs (Figure 5a) and for hollow MNPs (Figure 5b) at 5 K. A shift of the hysteresis loops toward both the negative field and the positive magnetization axis is observed, which, with an enhanced coercive field (H_C_^ZFC^ = 1099 Oe and H_C_^FC=10kOe^ = 1515 Oe of C/V/S and H_C_^ZFC^ = 601 Oe and H_C_^FC=10kOe^ = 636 Oe of hollow MNPs), constitutes strong evidence of the existence of an exchange bias effect [8,9].

In order to study the oleylamine coating effect of the C/V/S and hollow MNPs on reducing the direct interactions between MNPs, iron oxide solid MNPs of similar sizes and with an oleylamine coating were studied under FC conditions. Figure S2 shows a comparison between the hysteresis loops of the solid iron oxide MNPs (black points) and hollow MNPs (red points) under FC conditions (H_FC_ = 10 kOe) at 5 K. No EB effect on iron oxide solid MNPs was observed, suggesting that exchange interactions between MNPs are prevented with the oleylamine shell. Therefore, the effect of the interactions between MNPs in EB generation was satisfactorily avoided using a functional coating.

**Figure 6:**
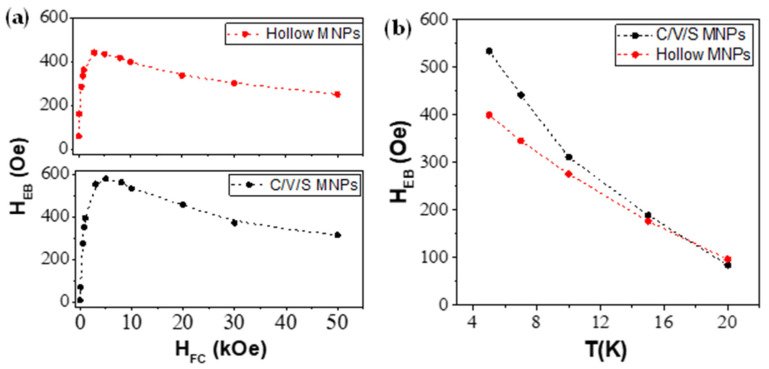
(**a**) Exchange bias field (H_EB_) measured at 5 K for core/void/shell (black dots) and hollow MNPs (red dots) as a function of the cooling fields (H_FC_). (**b**) Exchange bias field (H_EB_) for core/void/shell (black dots) and hollow MNPs (red dots) as a function of the temperature (T = 5–20 K).

Figure 6a shows the H_EB_ dependence with H_FC_. In comparison, the C/V/S MNPs exhibited a higher maximum H_EB_ (H_EB_ = 581.5 Oe) than the hollow MNPs (H_EB_ = 444.0 Oe). In addition, the maximum H_EB_ values were obtained at different cooling fields, H_FC_ = 5 kOe for C/V/S MNPs and H_FC_ = 3 kOe for hollow MNPs. In both MNPs, it was observed that the H_EB_ maximum was not reached in the largest applied cooling field (H_FC_ = 50 kOe), showing a maximum field below which H_EB_ decays. This trend was not observed in previous studies based on Fe/Fe_3_O_4_ MNPs of 13.8 nm, in which studies of the H_EB_ dependence with H_FC_ were carried out up to H_FC_ = 10 kOe [33]. In our case, this new trend was observed by investigating the H_EB_ behavior with cooling fields greater than 10 kOe (up to 50 kOe). In addition, the trend of H_EB_ with H_FC_ has not been reported in hollow MNPs due to the low value of H_EB_ [23]. Therefore, to our knowledge, the present study suggests a new trend of H_EB_ with H_FC_ in hollow iron oxide MNPs not reported so far.

Since EB is a phenomenon that occurs at low temperatures, the temperature dependence must be studied. Accordingly, magnetic measurements under FC conditions were carried out using a H_FC_ = 10 kOe and different temperatures, 5, 7, 10, 15, and 20 K. Figure 6b shows the H_EB_ dependence with temperature of C/V/S MNPs (black points) and hollow MNPs (red points). Both MNPs show a decrease in H_EB_ with temperature from the corresponding maximum values at 5 K (534.5 Oe for C/V/S and 400.0 Oe for hollow MNPs with H_FC_ = 10 kOe) to an approximate value of 100 Oe at 20 K. This shows that although H_EB_ is produced below the Néel temperature, the H_EB_ is drastically reduced with increasing temperature.

It has been reported that the morphology of MNPs plays an important role in EB generation. In core/shell MPNs, an exchange coupling between the core and shell components has been reported. A hysteresis loop shift has been observed in core/shell FeO/Fe_3_O_4_ MNPs with sizes between 10 and 35 nm and with surface-to-volume ratios between 0.60 and 0.17 [26,29,30,31]. In this case, the core features antiferromagnetic properties, while the shell features ferrimagnetic properties. As can be seen in Table 2, a direct relationship between the size of the particles and the H_EB_ value was not observed. Interestingly, an EB phenomenon was obtained in MNPs as large as 35 nm. This shows that EB is generated by the core–shell coupling because the size of the FeO/Fe_3_O_4_ MNPs is sufficiently high for the surface effects to not be noticeable. In addition, EB was reported in MNPs formed by a non-oxidized Fe core (ferromagnetic) and an Fe_3_O_4_ shell (ferrimagnetic) with D_T_ = 14 nm and δ_S_ = 2.5 nm [23]. Even though all these MNPs feature large H_EB_ values and EB generation is dominated by the core–shell, the transformation in hollow MNPs has not been studied. Therefore, core/void/shell MNPs formed prior to hollow MNP formation should be studied to better understand EB generation. These MNPs are formed by three surface layers, corresponding to the shell and the core. This leads to a higher surface-to-volume ratio and surface effects begin to play an important role. For this reason, it is important to note that the core/void/shell MNPs presented here feature a much higher surface-to-volume ratio (0.9) than the Fe/Fe_3_O_4_ MNPs shown in Table 2, even those with a smaller size (FeO/Fe_3_O_4_ MNPs of 10 nm with S/V = 0.6).

The hollow MNPs were only studied after the Fe core oxidation in the precursor C/V/S MNPs. However, the H_EB_ values obtained were significantly low and a study on the EB properties in C/V/S MNPs was not carried out. In addition, the H_EB_ value observed in these hollow MNPs is practically negligible [23,33] Here, a study of the EB effect in core/void/shell MNPs was carried out to understand which mechanisms affect EB generation in hollow MNPs.

On the other hand, a hysteresis loop shift in hollow γ-Fe_2_O_3_ MNPs (either a minor loop or an intrinsic EB effect) was reported (Table 3). The γ-Fe_2_O_3_ MNPs with a total diameter of D_T_ = 8.1 nm and shell thickness of δ_S_ = 1.6 nm showed a high hysteresis loop shift of 3000 Oe. However, since the maximum applied field is smaller than the irreversibility field, this shift is a minor loop and not an EB effect [37]. Similar behavior was reported in the γ-Fe_2_O_3_ MNPs with D_T_ = 9.2 nm and δ_S_ = 2 nm. In this case, the hysteresis loop shift was attributed to the high number of disoriented spins on the shell surface and at the interfaces of the crystallographic domains [34]. Furthermore, a minor loop effect was also observed in the γ-Fe_2_O_3_ MNPs with D_T_ = 9.4 nm and δ_S_ = 1.9 nm [32]. These three systems, with similar sizes and shell thicknesses, show that in smaller hollow MNPs, in which there is a greater number of disordered spins on the surface than in larger hollow systems, the hysteresis loops shift corresponds to a minor loop effect and not a EB phenomenon. A minor loop effect has also been reported in γ-Fe_2_O_3_ MNPs with a larger size (D_T_ = 14.7 nm and δ_S_ = 3.2 nm). In addition, due to surface effects, all these MNPs present low magnetization values.

In this work, iron oxide-based hollow MNPs with D_T_ = 13.2 nm and δ_S_ = 4.5 nm and with surface-to-volume values of 0.6 are presented, showing an intrinsic EB phenomenon with enhanced magnetization. To this end, a complete study of the role played by interfacial and surface spins in the EB generation must be carried out both in hollow MNPs and in the precursor C/V/S MNPS prior to the complete oxidation of the Fe core.

### 3.4. Role of Surface Spins

**Figure 7:**
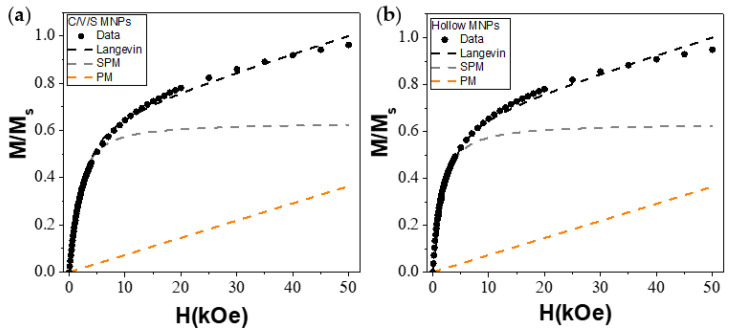
Initial magnetization curve at 300 K of (**a**) core/void/shell and (**b**) hollow iron oxide MNPs fitted to Equation (3). The black, gray, and orange dashed curves represent the Langevin fit and the SPM and PM contributions, respectively.

In order to determine the role of surface spins in the magnetic properties of MNPs, the superparamagnetic (SPM) and paramagnetic (PM) contributions of the MNPs were studied. The experimental data are fitted according to the Langevin function with an added linear term corresponding to the PM contribution:

(3) $$M\left(H\right)={\;M}_{S}^{\mathrm{SPM}}\left[\cot h\left(\frac{\mu \;H}{K_{B}\;T}\right)-{\left(\frac{\mu \;H}{K_{B}\;T}\right)}^{-1}\right]+C^{\mathrm{PM}}H\;$$

where M_S_^SPM^ is the saturation magnetization of the SPM component, μ is the average magnetic moment of the MNPs, C^PM^ is the susceptibility of the paramagnetic contribution, and H is the magnetic field. Figure 7a,b shows the initial magnetization at 300 K of C/V/S and hollow MNPs, respectively.

As can be seen from the experimental data (black points) and the Langevin fit (black dashed line) of the C/V/S and hollow MNPs, respectively, the SPM contribution (gray dashed line), corresponding to the inner spins, and the PM contribution (orange dashed line), corresponding to the surface spins, were obtained. From these fits, SPM contributions of 76 and 73% for C/V/S and hollow MNPs, respectively, were obtained. On the other hand, PM contributions represented 23% and 26% for C/V/S and hollow MNPs, respectively.

From these results, it is possible to conclude that a significant number of surface spins in both MNPs play an important role in the magnetic properties. In turn, the greater number of surface spins present in the hollow MNPs (26%) may be due to the larger shell size of the hollow MNPs. At low temperatures, these spins may enter a spin-glass-like state, whose exchange coupling with the ordered spins generates an EB effect. The spin-glass-like behavior was inferred from the AC measurements by the Φ parameter. Values of Φ below 0.06 demonstrate spin-glass-like behavior in MNPs [32]. Values of 0.04 and 0.05 were obtained for the core/void/shell and hollow MNPs, respectively, showing spin-glass behavior (See Supporting Information).

However, the higher surface-to-volume ratio of the C/V/S (S/V = 0.9) compared to the hollow MNPs (S/V = 0.6) indicates a greater surface area due to the additional surface area of the Fe core. This suggests the greater role of the surface spins located in the shell in the magnetic properties of the MNPs than the role of the surface spins located in the Fe core, which were negligible. Based on the surface area of the inner and outer surfaces of the hollow MNPs, an estimation of the % of surface spins corresponding to each surface was carried out. Values of 21.8 and 4.2% for the external and internal surface spins, respectively, were obtained in the hollow MNPs, showing the significant contribution of the surface spins of the external layer. In addition, the effect of the inner surface spins was studied by comparing the EB phenomenon in the solid and hollow MNPs. Figure S2 shows a comparison between the hysteresis loops of the solid iron oxide MNPs (black points) and the hollow MNPs (red points) under FC conditions (H_FC_ = 10 kOe) at 5 K. A significant reduction in the PM contribution was observed in the solid MNPs. Considering the similar amount of surface spins in the external layer in both MNPs, the importance of the additional internal surface spin layer in the magnetic properties and in the EB generation was demonstrated. Although H. Khurshid et al. [34] have discussed the effect of surface internal spins in this way, the multiple phenomena in EB generation, such as the exchange coupling of frozen spins from internal nanograins, do not allow a clear differentiation between the internal and external spins’ contribution in EB. Therefore, more experimental studies must be carried out.

In a similar study, the contribution of the SPM and PM components was obtained in 9.2 nm and 18.7 nm γ-Fe_2_O_3_ hollow MNPs. In this case, PM contributions of 87% (9.2 nm) and 3% (18.7 nm) were obtained. An increase in the linear component of magnetization (and a greater number of surface spins) was demonstrated due to the reduction in the thickness of the shell [34]. For this reason, a lower PM contribution was observed on the magnetic properties of Fe/Fe_3_O_4_ MNPs and hollow Fe_3_O_4_ MNPs of 13.8 and 16.0 nm, respectively, by Ong et al. [33] than in our MNPs of smaller sizes (11.9 nm for C/V/S and 13.2 nm for hollow MNPs). Thus, the significantly lower values obtained in the 16.0 nm hollow Fe_3_O_4_ MNPs (H_EB_ = 133 Oe) than in our hollow MNPs (H_EB_ = 444 Oe) highlight the importance of surface spins to the improvement the EB properties of hollow iron oxide MNPs.

### 3.5. Remanence (M_R_) and Coercivity (H_C_) Dependence with Cooling Field (H_FC_)

**Figure 8:**
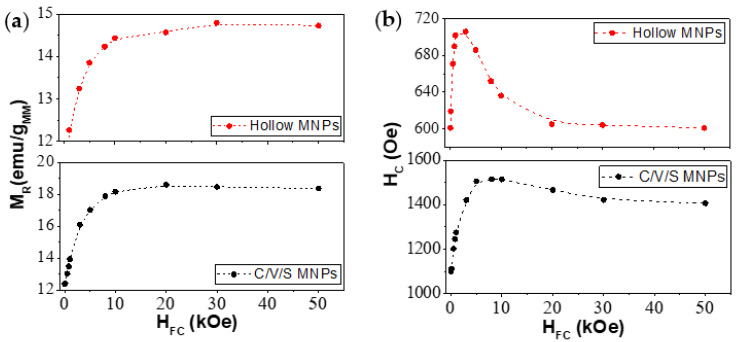
(**a**) Remanent magnetization (M_R_) and (**b**) coercivity (H_C_) measured at 5 K for core/void/shell (black dots) and hollow MNPs (red dots) as a function of the cooling field (H_FC_).

A study of the remanence (M_R_) and coercive force (H_C_) dependence with H_FC_ was carried out with the aim of understanding the EB generation process. Figure 8a shows the M_R_ dependence with H_FC_ of the C/V/S MNPs (black dots) and hollow MNPs (red dots). For small fields, M_R_ increases with H_FC_. However, from H_FC_ ≥ 20 kOe, a maximum value was reached, and M_R_ remained constant. This indicates that the sample retained more magnetization by increasing H_FC_. Figure 8b shows the H_C_ dependence with H_FC_ of C/V/S MNPs (black dots) and hollow MNPs (red dots). Similarly to M_R,_ a steep rise in H_C_ along with increasing H_FC_ from low values was observed. Next, a maximum value of H_C_ was reached, followed by a decrease for larger H_FC_. The maximum H_C_ was reached at different H_FC_ for each MNP: 5 kOe for the C/V/S MNPs and 3 kOe for the hollow MNs.

Two different regions were observed in the M_R_, H_C,_ and H_EB_ dependence with H_FC_. In the first region, for low H_FC_, where M_R_, H_C,_ and H_EB_ increase with H_FC_. A preferential direction, along which the magnetic moments tended to freeze at 5 K, was induced by the cooling field. Therefore, the exchange anisotropy was not averaged out (as in ZFC conditions) and a net exchange bias effect was observed. However, for high H_FC_, the coupling between the magnetic moments and the H_FC_ (Zeeman coupling) was no longer negligible compared to the exchange coupling existing in the MNPs. Thus, the energy associated with the exchange interactions was not minimized due to Zeeman coupling, generating a decrease in H_C_ and H_EB_ [42]. This Zeeman coupling was greater as H_FC_ increased. It can be seen how the maximum of H_EB_ was different in both MNPs, suggesting that the Zeeman coupling begins to be noticeable at lower H_FC_ in hollow MNPs. In the hollow MNPs, the first region in which M_R_, H_C_, and H_EB_ increased with H_FC_ was produced for H_FC_ < 3 kOe and in the C/V/S MNPs for H_FC_ < 5 kOe. This suggests that the effect of averaging of the anisotropy, due to randomness, was more reduced in the C/V/S than in the hollow MNPs.

### 3.6. Frozen Spins Behavior

In order to understand the behavior of the exchange coupling in both MNPs and their dependence on H_FC_, a study of the number of frozen spins in the MNPs was carried out. A vertical shift in the hysteresis loop under FC conditions in the direction of the cooling field is proportional to the number of frozen spins that cannot be reversed by the measurement field [53]. The net moment of frozen spins can be quantified as:

(4) $$M_{f}=\frac{1}{2}\;\left[M\left(H^{+}\right)-M\left(H^{-}\right)\right]\;$$

Here the positive direction is the direction of the cooling field. M_f_ studies were carried out using different H_FC_ and different temperatures of both C/V/S and hollow MNPs to understand the role of frozen spins in the EB generation.

**Figure 9:**
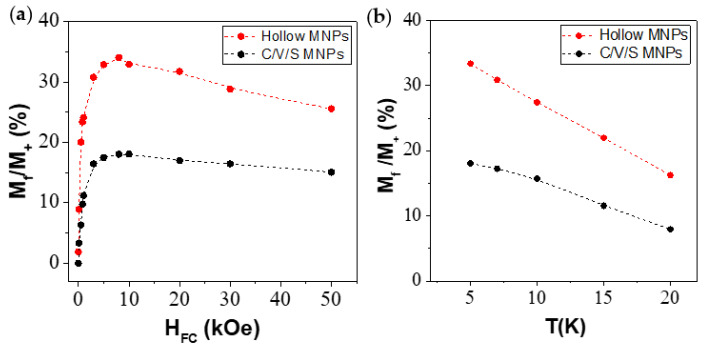
(**a**) Relative magnitude of frozen moment (M_f_/M(H^+^)) measured at 5 K for C/V/S (black dots) and hollow MNPs (red dots) as a function of the cooling field (H_FC_). (**b**) Relative magnitude of frozen moment (M_f_/M(H^+^))) for C/V/S (black dots) and hollow MNPs (red dots) as a function of the temperature (T = 5–20 K).

Figure 9a shows the relative magnitude of the frozen moment (M_f_/M(H^+^)) for the C/V/S MNPs (black points) and hollow MNPs (red points). Similar trends in both MNPs were observed. The number of frozen spins showed a drastic increase as the value of the cooling field increased. Subsequently, the number of spins reached a maximum value that did not correspond to the H_FC_. Once this maximum was reached, the number of frozen spins began to decrease. This fact indicates that the irreversible spins began to reverse their moment due to the high magnetic field applied. This fact agrees with the trends observed for H_C_ and H_EB_, whose values reached a maximum due to the Zeeman coupling. The M_f_/M(H^+^) maximum was 18% (for H_FC_ = 10 kOe) and 34% (for H_FC_ = 8 kOe) for the C/V/S MNPs and hollow MNPs, respectively, demonstrating the presence of exchange coupling in both MNPs. The core/void/shell MNPs did not present a direct exchange coupling, but rather discontinuously, due to the presence of gaps between the core and the shell; it must be considered that exchange coupling is a short-range interaction and decays exponentially with distance [33]. However, exchange interactions between the Fe core and the iron oxide shell cannot be totally ruled out. On the other hand, the hollow MNPs did not present said Fe core and the presence of frozen spins cannot be explained in this way. This result, together with the HRTEM images, allows us to show that the shell of the C/V/S and the hollow MNPs was formed by nanograins that were coupled to each other. This coupling was, in part, responsible of the EB generation in the hollow MNPs and it is presented as an alternative to classic EB generation, which is produced by two magnetically different materials. The higher number of frozen spins in the hollow MNPs may have been due to the larger size of the shell (3.7 nm for the hollow MNPs and 2.4 nm for the C/V/S MNPs) and, therefore, of the nanograins that formed the shell. In addition, the presence of the Fe core in the C/V/S MNPs formed by reversible spins must be considered. This number of reversible spins present in the Fe core could counteract the frozen spins in C/V/S MNPs and, therefore, the percentage of frozen spins in C/V/S MNPs was lower.

Figure 9b shows the temperature dependence of (M_f_/M(H^+^)) for the C/V/S MNPs (red points) and hollow MNPs (black points). In the hollow MNPs, a decrease from 33 to 16% was observed when the temperature increased from 5 to 20 K. On the other hand, the C/V/S MNPs showed a decrease from 18 to 8% at the same temperatures. This decrease was also observed in H_EB_, showing the importance of interfacial spins in EB generation.

**Figure 10:**
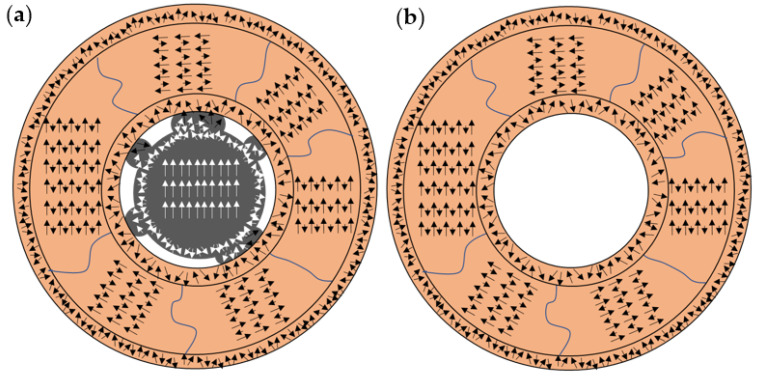
Schematic illustration of the different spin arrangements in (**a**) core/void/shell and in (**b**) hollow MNPs, with inner magnetic domains, external surfaces (inner and outer), and core magnetic domain.

Based on these results, a spin distribution for both MNPs is proposed, with the aim of shedding light on EB generation. Figure 10 shows a diagram of the spin distribution of (a) C/V/S and (b) hollow MNPs. The presence of two layers of surface spin in the shells of the MNPs is suggested. From the percentage of surface spins (23% for C/V/S and 26% for hollow MNPs), a similar distribution and effect on EB generation in these spins in both MNPs can be inferred. The surface spin contribution in EB generation, when entering a spin-glass-like state, should not be ignored, due to the high values obtained. Compared to the work reported by Ong et al. [23], a key role of surface spins to inducing an improvement in H_EB_ was observed. Furthermore, the results obtained from the frozen spins show a nanograin structure in the shell of the MNPs whose spins were coupled to each other. Due to the presence of the Fe core reversible spins, a lower percentage of frozen spins in C/V/S MNPs was obtained. However, the EB generation process due to the frozen spins of the nanograins must be similar in both MNPs. The significant difference resides in the presence of the Fe core. In the C/V/S MNPs, there was a discontinuous coupling between the Fe (FM) core and the iron oxide (FI) shell, which was responsible for the higher H_EB_ values obtained in the C/V/S MNPs. However, this coupling did not dominate in the generation of EB, since the H_EB_ values obtained were not significantly higher.

## 4. Conclusions

In summary, we presented the study of the intrinsic EB effect on iron oxide (with Fe_3_O_4_ or γ-Fe_2_O_3_ as the main magnetic phase) hollow MNPs (H_EB_ = 444 Oe) with improved magnetization (50 emu/g) within an extended range of H_FC_ (from 0.1 to 50 kOe). The number of surface spins present in the hollow MNPs and in the precursors with core/void/shell structure were studied. The important role of the surface spins in the magnetic properties of MNPs and, in turn, in the enhancement of H_EB_ in hollow MNPs, was inferred. On the other hand, the dependence of frozen spins on the cooling field and temperature was measured and found to be similar to the relationship between H_EB_ with H_FC._ This highlighted the notable influence of frozen spins on EB generation. Moreover, the relationship between H_EB_ and a large interval of cooling fields revealed the maximum H_EB_ values at particular H_FC_ and showed new trends, not reported so far, in iron-oxide-based hollow MNPs. Therefore, this finding can be useful in the design of hollow iron oxide MNPs with H_EB-max_ and H_FC-max_ for in-demand applications.

## Figures and Tables

**Table 1:** Inner radius (R_outer_), outer radius (R_inner_), core radius (R_Core_), shell thickness (δ_shell_), void thickness (d_void_) and surface-to-volume ratio (S/V) values of MNPs.

	R_outer_ (nm)	R_inner_ (nm)	R_Core_ (nm)	δ_shell_ (nm)	δ_void_ (nm)	S/V
Hollow	6.6	2.9	-	3.7	5.8	0.6
Core/void/shell	6.0	3.6	2.3	2.4	1.3	0.9

**Table 2:** Total diameter (D_T_), shell thickness (δ_S_), hysteresis loop shift (H_EB_), and surface-to-volume ratio(S/V) values of iron oxide-based core/shell MNPs.

Core/Shell	Void?	d_T_ (nm)	δ_S_ (nm)	H_EB_ (Oe)	S/V	Ref
FeO/Fe_3_O_4_	No	10.1	0.6	1700	0.60	[26]
FeO/Fe_3_O_4_	No	14	3.5	471	0.43	[31]
FeO/Fe_3_O_4_	No	18	~6	~1000	0.33	[46]
FeO/Fe_3_O_4_	No	35	4	2260	0.17	[30]
Fe/Fe_3_O_4_	No	13.8	2.5	1190	0.43	[23]
C/V/S MNPs	Yes	11.9	2.4	581.5	0.9	Present Study

**Table 3:** Total diameter (D_T_), shell thickness (δ_S_), hysteresis loop shift values (H_EB_), type of hysteresis loop shift, and surface-to-volume ratio (S/V) of iron-oxide-based hollow MNPs.

Hollow	d_T_ (nm)	δ_S_ (nm)	H_EB_ (Oe)	Type	S/V	Ref
γ-Fe_2_O_3_	8.1	1.6	~3000	Minor Loop	1.29	[37]
γ-Fe_2_O_3_	9.2	2	-	Minor Loop	1.05	[34]
γ-Fe_2_O_3_	9.4	1.9	~5000	Minor Loop	1.10	[32]
γ-Fe_2_O_3_	14.7	3.2	~7000	Minor Loop	0.66	[32]
γ-Fe_2_O_3_	18.7	4.5	960	EB	0.47	[34]
Fe_3_O_4_	16.0	4.5	133	EB	0.49	[23]
Iron Oxide	13.2	3.7	444	EB	0.6	Present Study

## Data Availability

Not applicable.

## Supplementary Materials

The following are available online at https://www.mdpi.com/article/10.3390/nano12030456/s1. Figure S1: Temperature dependence of the real component χ’(T) of (**a**) C/V/S MNPs and (**b**) hollow MNPs at 1 Oe. Insets show the imaginary component χ’’(T) of the magnetic susceptibility of C/V/S MNPs and hollow MNPs; Figure S2: Hysteresis loops of solid iron oxide based MNPs (black points) and hollow iron oxide based MNPs (red points) at 5 K and with H_FC_ = 10 kOe in the range of (**a**) −50 and 50 kOe. For clarity the range between −5 and 5 kOe is shown in (**b**).
