# Supplementary material for: Significant Surface Spin Effects and Exchange Bias in Iron Oxide-Based Hollow Magnetic Nanoparticles
Source: Nanomaterials (Basel). 2022 Jan 28;12(3):456. doi: 10.3390/nano12030456 (PMC8838860; doi:10.3390/nano12030456)
Supplement: Supplementary file 1 [file nanomaterials-12-00456-s001.zip › nanomaterials-1473724-supplementary.pdf]

nanomaterials

MDPI

Supporting Information

Significant Surface Spin Effects and Exchange Bias in Iron Oxide-Based Hollow Magnetic Nanoparticles

Pelayo García Acevedo [1,*], Manuel A. González Gómez [1], Ángela Arnosa Prieto [1], Jose S. Garitaonandia [2], Yolanda Piñeiro [1] and José Rivas [1,*]

[1] NANOMAG Laboratory, Applied Physics Department, iMATUS Materials Institute, Universidade de Santiago de Compostela, 15782 Santiago de Compostela, Spain; manuelantonio.gonzalez@usc.es (M.A.G.G.); angela.arnosa@usc.es (Á.A.P.); y.pineiro.redondo@usc.es (Y.P.)

[2] Faculty of Science and Technology, University of the Basque Country (UPV/EHU), 48940 Leioa, Spain; js.garitaonandia@ehu.eus

* Correspondence: pelayo.garcia.acevedo@usc.es (P.G.A.); jose.rivas@usc.es (J.R.); Tel.: +34-881813097 (P.G.A.); +34-881813062 (J.R.)

1. Spin-Glass-like Behaviour

The magnetic dynamics for core/void/shell (C/V/S) MNPs and hollow MNPs were studied using real χ′(T) and imaginary χ′′(T) components of the AC susceptibility. Figures S1 shows the temperature dependence of the real part χ′(T) (figure S1.a for C/V/S MNPs and Figure S1b for hollow MNPs) and the imaginary part χ′′(T) (Inset in figure S1.a for C/V/S MNPs and inset in figure S1.b for hollow MNPs) of the ac susceptibility at different frequencies (10, 200, 500 and 1000 Hz). These curves show a maximum for both χ′(T) and χ′′(T) components, defined as T'_{max} and T''_{max}. For χ′(T) component, a shift to higher temperatures when the frequency ω increases is observed, while the value of χ′ decreases. It is typical in disordered systems such as spin glasses [1]. However, χ′(T) is frequency independent at $T>T'_{max}$, indicating a superparamagnetic behaviour for both MNPs. For χ′′(T) component, a shift to higher temperatures is also observed, but the value of χ′′ increases. The blocking temperature, T_B, is obtained as the temperature where the maximum happens in the χ′(T) curve. The T_B values obtained are 111.61 K (ω = 10 Hz), 118.72 K (ω = 200 Hz), 119.61 K (ω = 500 Hz) and 120.50 K (ω = 1000 Hz) for C/V/S MNPs and 100.00 K (ω = 10 Hz), 108.88 K (ω = 200 Hz), 109.77 K (ω = 500 Hz) and 111.55 K (ω = 1000 Hz) for hollow MNPs.

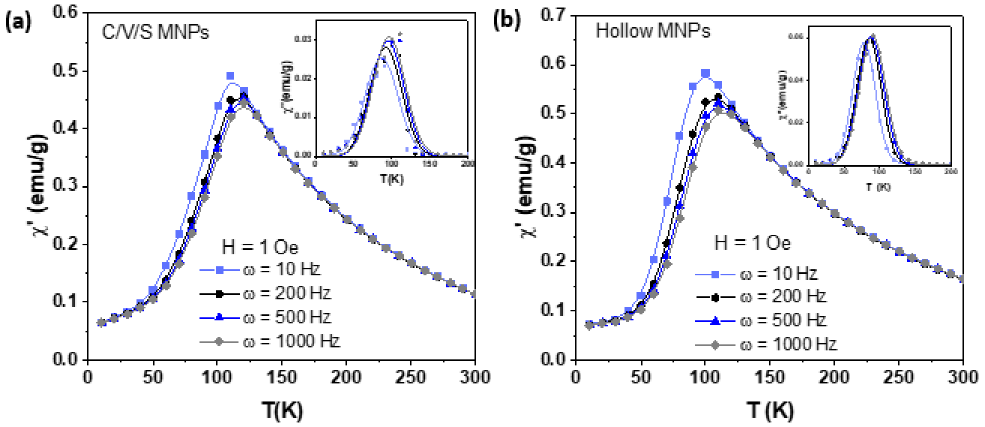

Figure S1. Temperature dependence of the real component χ′(T) of (**a**) C/V/S MNPs and (**b**) hollow MNPs at 1 Oe. Insets show the imaginary component χ′′(T) of the magnetic susceptibility of C/V/S MNPs and hollow MNPs**.**

From AC measurements the spin-glass-like behavior of MNPs was obtained using the ϕ parameter from the relation [1]:

$$\Phi = \frac{\Delta T'_{max}}{T'_{max} \cdot \Delta\log(\omega)} \quad \text{(S1)}$$

Where $\Delta T'_{max}$ is the difference between the maximum values of T'_{max} in an interval $\Delta\log(\omega)$. Depending on the values obtained from Φ, it is possible to differentiate whether the MNPs behave as a spin-glass-like system (if $\Phi < 0.06$) [2]. In our case, values of $\Phi = 0.04$ were obtained for C/V/S MNPs and $\Phi = 0.05$ for hollow MNPs, suggesting a spin-glass-like surface behavior in both MNPs.

2. Hysteresis Loops under FC Conditions of Solid and Hollow Fe_3O_4 MNPs

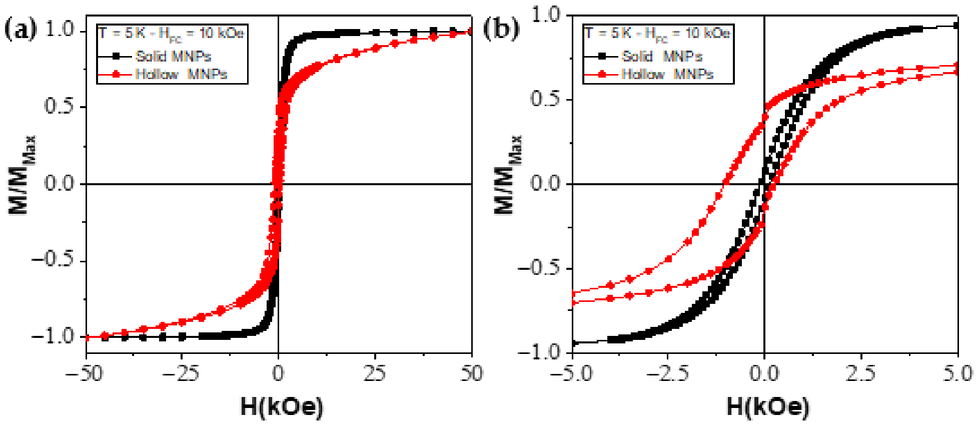

Figure S2. Hysteresis loops of solid iron oxide based MNPs (black points) and hollow iron oxide based MNPs (red points) at 5 K and with H_{FC} = 10 kOe in the range of (**a**) −50 and 50 kOe. For clarity the range between −5 and 5 kOe is shown in (**b**).

References

1. Masunaga, S.H.; Jardim, R.F.; Fichtner, P.F.P.; Rivas, J. Role of dipolar interactions in a system of Ni nanoparticles studied by magnetic susceptibility measurements. *Phys. Rev. B* **2009**, *80*, 184428, https://doi.org/10.1103/physrevb.80.184428.
2. Nemati, Z.; Khurshid, H.; Alonso, J.; Phan, M.H.; Mukherjee, P.; Srikanth, H. From core/shell to hollow Fe/γ-Fe2O3nano-particles: evolution of the magnetic behavior. *Nanotechnology* **2015**, *26*, 405705, https://doi.org/10.1088/0957-4484/26/40/405705.
